# CXCL9 recombinant adeno-associated virus (AAV) virotherapy sensitizes glioblastoma (GBM) to anti-PD-1 immune checkpoint blockade

**DOI:** 10.21203/rs.3.rs-3463730/v1

**Published:** 2023-11-14

**Authors:** Christina von Roemeling, Oleg Yegorov, Changlin Yang, Kelena Klippel, Rylynn Russell, Vrunda Trivedi, Alisha Bhatia, Bently Doonan, Savannah Carpenter, Daniel Ryu, Adam Grippen, Hunter Futch, Yong Ran, Lan Hoang-Minh, Frances Weidert, Todd Golde, Duane Mitchell

**Affiliations:** University of Florida; University of Florida; University of Florida; University of Florida; University of Florida; Stanford University; University of Florida; University of Florida; University of Florida; Emory University; MD Anderson Cancer Center; Emory University; Emory University; University of Florida; Department of Neurosurgery, Preston A. Wells, Jr. Center for Brain Tumor Therapy, University of Florida; Emory University; University of Florida

## Abstract

The promise of immunotherapy to induce long-term durable responses in conventionally treatment resistant tumors like glioblastoma (GBM) has given hope for patients with a dismal prognosis. Yet, few patients have demonstrated a significant survival benefit despite multiple clinical trials designed to invigorate immune recognition and tumor eradication. Insights gathered over the last two decades have revealed numerous mechanisms by which glioma cells resist conventional therapy and evade immunological detection, underscoring the need for strategic combinatorial treatments as necessary to achieve appreciable therapeutic effects. However, new combination therapies are inherently difficult to develop as a result of dose-limiting toxicities, the constraints of the blood-brain barrier, and the suppressive nature of the GBM tumor microenvironment (TME). GBM is notoriously devoid of lymphocytes driven in part by a paucity of lymphocyte trafficking factors necessary to prompt their recruitment, infiltration, and activation. We have developed a novel recombinant adeno-associated virus (AAV) gene therapy strategy that enables focal and stable reconstitution of the GBM TME with C-X-C motif ligand 9 (CXCL9), a powerful call-and-receive chemokine for cytotoxic T lymphocytes (CTLs). By precisely manipulating local chemokine directional guidance, AAV-CXCL9 increases tumor infiltration by CD8-postive cytotoxic lymphocytes, sensitizing GBM to anti-PD-1 immune checkpoint blockade (ICB). These effects are accompanied by immunologic signatures evocative of an inflamed and responsive TME. These findings support targeted AAV gene therapy as a promising adjuvant strategy for reconditioning GBM immunogenicity given its excellent safety profile, TME-tropism, modularity, and off-the-shelf capability, where focal delivery bypasses the constrains of the blood-brain barrier, further mitigating risks observed with high-dose systemic therapy.

## Introduction

Breakthroughs in immunotherapy including ICB, monoclonal and bispecific antibodies, and CAR T cell therapy, have ignited the hope of achieving durable remission in even the most recalcitrant tumors. Although these strategies are capable of producing remarkable responses, therapeutic benefit is seen in only a small proportion of patients with many proposed reasons for lack of response^1^. Presently, T cell infiltration and abundance within the tumor microenvironment is one of the most predictive biomarkers for response to immunotherapy ^2–4^. Unfortunately, diseases like GBM and many other solid tumors demonstrate low baseline infiltration of lymphocytes that is only marginally improved by treatment^5,6^. For GBM patients in particular, generalized lymphopenia as a result of impaired lymphocyte egress^7^ alongside the lymphodepleting nature of conventional treatment further detracts from successful adaptive immune recognition of these tumors^8^. Preclinical studies on cellular therapy imaging and trafficking show that a comparably lower fraction of T cells can be found in these brain tumors as compared to lung, liver, and spleen tissues in comparative mouse models^9,10^. Similar hurdles with respect to CAR T cell penetration into brain tumors in the clinical setting have necessitated alternative locoregional delivery strategies^11^. Together these observations indicate that ineffective T cell migration and infiltration into GBM tumors may represent a principal barrier to immunotherapy. Solving this problem requires both an improved understanding of the chemical signals that govern T cell chemotaxis into tumors and identifying a method to amplify those signals.

CTLs are recruited via long-range signaling mediated by the diffusion of chemokines present in inflammatory environments. During glioma formation, tumors manufacture immune-suppressive chemokines and cytokines that co-opt resident cells, resulting in the preferential recruitment of immune suppressor cells from the periphery ^12^. Additionally, we have found that primary patient glioma samples are deficient in lymphocyte-specific chemokines. To overcome this problem, we propose the use of *in situ* recombinant AAV expressing a lymphocyte trafficking chemokine payload to restore expression of lymphocyte chemotaxis. AAV vectors are the leading platform for gene delivery due to their efficacy, ease of use, and safety profile^13^. Additionally, multiple AAV gene therapies have reached FDA approval for a variety of diseases^13,14^. Thus, AAV vectors are an ideal platform for transforming targeted cell populations while minimizing potential patient risk. Following a comprehensive chemokine screen of clinical tumor specimens, we have identified CXCL9 as a candidate lymphocyte call-and-receive signal absent in GBM. We examined AAV delivered CXCL9 transgene tropism, durability, and impact on CTL tumor migration. Using syngeneic preclinical model systems of GBM, we further evaluated the therapeutic benefit of our AAV gene therapy alongside anti-PD-1 ICB. Single cell RNA sequencing (scRNAseq) of immune cells isolated from tumors during treatment reveal widespread immunological reconditioning of tumors, with improved effector lymphocyte recruitment that yields long-term survival outcomes in aggressive preclinical GBM models. This work suggests direct conditioning of the tumor microenvironment by AAV-CXCL9 constructs could potentially overcome a key resistance factor in GBM, sensitizing tumors to immune mediated therapies.

## RESULTS

### Chemokine analysis of Human GBM favors MDSC recruitment over lymphocyte infiltration

**Figure 1.**
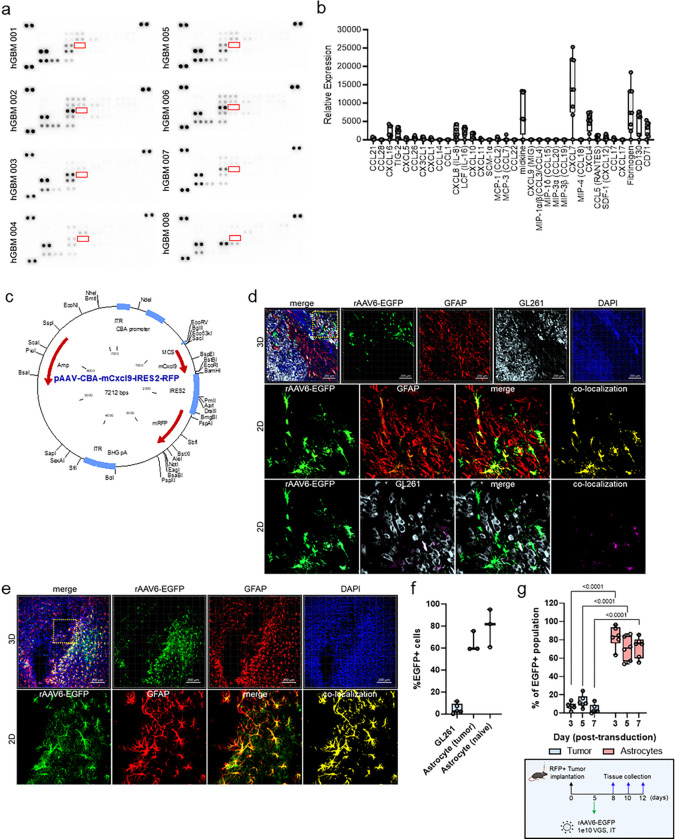
Chemokine signature of glioblastoma tumors. (a) Immunoblots of GBM samples showing signal intensity of 31 chemokines (n=8). CXCL9 (undetected) is outlined in red. (b) Cumulative relative protein expression of immunoblots shown in panel (a). (c) Recombinant AAV6 vector design encoding CXCL9 and the fluorescent reporter gene RFP. (d) 3D IHC of RFP-labeled GL261 tumor tissue collected 1 week following AAV6-EGFP injection. The top row depicts 3D rendering of tissue captured at 10x magnification. AAV6 transduced cells are shown in green pseudocolor, GFAP in red pseudocolor, RFP+ tumor cells in light blue pseudocolor, and DAPI nuclear stain in dark blue pseudocolor. 2^nd^ and 3^rd^ rows depict 2D digital zoom as outlined by the yellow dashed line in the top row to enhance cellular resolution. Voxel-based co-localization performed using Imaris imaging software between AAV6 and GFAP (2^nd^ row) and AAV6 and tumor cells (3^rd^ row) is shown as a separate channel (yellow or pink pseudocolor). Representative images selected from a minimum of 3 replicates. (e) 3D IHC of AAV6-EGFP transduction in age-matched naïve control mice. The top row depicts 3D rendering of tissue captured at 10x magnification. AAV6 transduced cells are shown in green pseudocolor, GFAP in red pseudocolor, and DAPI nuclear stain in dark blue pseudocolor. 2^nd^ row depicts 2D digital zoom as outlined by the yellow dashed line in the top row to enhance cellular resolution. Voxel-based co-localization performed using Imaris imaging software between AAV6 and GFAP is shown as a separate channel (yellow pseudocolor). Representative images selected from a minimum of 3 replicates. (f) Quantitative summary of voxel-based AAV6 co-localization with either tumor (GL261, n=5) or astrocytes in each tumor-bearing (n=3) and naïve mice (n=3). Values are presented as the cumulative mean ± standard deviation. (g) Box-whisker plot of flow cytometry quantitation of AAV6 (EGFP+) co-localization with either tumor cells (GL261, RFP+) or astrocytes (GFAP+, RFP−) at 3-, 5-, and 7-days post AAV6 transduction as illustrated in the schematic below. Two-way ordinary ANOVA statistical analysis performed comparing percent transduction between tumor and astrocytes across matched time points, n=6 per time point. P-values = or < 0.05 are considered statistically significant.

Upon examination of human glioma tumors for CD3 protein expression provided through the Human Protein Atlas, we found that > 80% of tumor specimens evaluated are negative for lymphocyte infiltrates (**Extended Data** Fig. 1a), corroborating prior literature describing the lymphocyte-replete nature of these tumors^15^. To identify if a paucity of lymphocyte chemotactic factors is a contributing aspect, human glioma samples were screened via chemokine proteome array. Of these, CXCL4, CXCL7, CXCL8 (IL-8), CXCL16, LCF (IL-16), TIG-2, and midkine (MDK) emerged as the most abundant secreted chemokines detected (Fig. 1a,b), where these ligands play a significant role in recruiting myeloid-derived suppressor cells in the context of gliomagenesis^16^. Notably, chemotactic factors that favor lymphocyte recruitment were poorly expressed, including CXCL9 (MIG) and MIP-1α/β (CCL3/CCL4) (Fig. 1a,b). CXCL9 is a powerful attractant known to induce the migration, differentiation, and activation of CTLs^17^. Expression of CXCL9 has been shown to correlate with anti-tumor immune activity and is predictive of response to ICB in several solid tumors^18^. We hypothesized that AAV delivery and restoration of CXCL9 as a “call-and-receive” signal for T lymphocytes within the TME would enhance their recruitment and infiltration in the tumor.

Historically, targeted transduction of cancer with AAV has proven challenging despite elegant efforts in capsid evolution studies and capsid engineering. To achieve sufficient transgene expression, transduction of either tumor cells or tumor-associated stroma is likely necessary, where intra-tumoral delivery would minimize the potential for systemic toxicities and decrease off target homing of T cells. To identify an appropriate AAV capsid for targeting glioma tumors, we performed an *in vitro* capsid screen. Enhanced green fluorescent protein (EGFP) was encoded into an AAV2 single stranded vector, utilizing the non-cell-autonomous, constitutively active CBA promoter to drive transgene expression (AAVn-EGFP). These constructs were pseudotyped into 29 unique capsids as previously described^19^. Transduction in 15 unique glioma models, including primary human^20^ and murine xenografts, was assessed via EGFP relative fluorescence expression (**Extended Data** Fig. 1b). AAV6 was selected for further examination as it demonstrates moderate to high transduction in nearly all models tested and is further substantiated by excellent CNS transduction in other studies^19,21^. AAV6 capsids encoding each CXCL9, EGFP, blue fluorescent protein (BFP), and empty vector control (Fig. 1c, **Extended Data** Fig. 1c,d,e) were designed for further testing. Quantitative flow cytometric evaluation of EGFP expression in three distinct syngeneic murine GBM models 72 hours following AAV6-EGFP transduction shows moderate transduction of GL261 and KR158, with > 25% of cells positive for the transgene at this time point, with lower transduction observed in CT-2A cells (< 20%) (**Extended Data** Fig. 1f).

### AAV6 transduces tumor-reactive astrocytes in vivo in preclinical models of GBM

While we demonstrate good targeting of glioma cells in an *in vitro* setting, AAV6 has also been reported capable of targeting other cell populations in the CNS^19,21^. These studies have largely been performed in the context of naïve mice or in models of neurodegenerative disease, which may or may not be directly applicable to CNS malignancy. To define AAV6 tropism in murine GBM *in vivo*, AAV6-EGFP (**Extended Data** Fig. 1c) was intratumorally injected into established intracranial KR158 and GL261 tumors. EGFP expression was detected 1 week following tumor transduction in both models (**Extended Data** Fig. 2a,b), however the morphological appearance and contiguous distribution of transduced cells suggest that AAV6 targeted cells are likely tumor-associated, and not cancer cells directly. Microglia and tumor-associated macrophages are reported to comprise a significant cellular proportion of glioma tumors^22^, and so we sought to identify if AAV6 was targeting either population. KR158 and GL261 were implanted into CCR2^RFP^CX3CR1^GFP^ (B6.129(Cg)-*Cx3cr1*^tm1Litt^ *Ccr2*^tm2.1lfc^/JernJ) dual reporter mice, where microglia can be identified via GFP expression, and bone marrow-derived inflammatory cells via RFP expression^23,24^. Tumors were evaluated by 3D IHC for viral transduction one week following intratumor injection with AAV6 encoding a BFP reporter (**Extended Data** Fig. 1d). In both tumor model systems, we found no co-localization between BFP and either RFP or GFP, indicating that neither microglia nor tumor-associated macrophages are the principal target of AAV6 transduction (**Extended Data** Fig. 3a,b). To assess the degree of AAV6 transduction specifically in tumor cells we implanted mice with RFP-labeled KR158 or GL261 cells. Tumors were evaluated by 3D IHC for viral transduction one week following intratumor injection with AAV6-EGFP. Resected tumors were immuno-labeled against glial fibrillary acidic protein (GFAP) to detect astrocytes, another candidate tumor-associated cell population. Both tumor models reveal a high degree of overlap between GFAP (red pseudocolor) and EGFP (green pseudocolor), with minimal overlap between tumor cells (light blue pseudocolor) and EGFP (Fig. 1d, **Extended Data** Fig. 3c), indicating that EGFP-positive cells are likely astrocytes. Voxel-based co-localization algorithms to quantitate EGFP co-localization with each tumor or astrocytes confirm astrocytes as the principal cell target of AAV6 transduction, accounting for ~ 60–70% of EGFP-positive cells in both GL261 (Fig. 1e) and KR158 (**Extended Data** Fig. 3d) intracranial tumors. Because tumor presence can stimulate different activation states in astrocytes that may cause them to be more or less susceptible to viral transduction^25,26^, we also evaluated CNS tropism of AAV6 in age-matched naïve mice. AAV6 was equally efficient at transducing astrocytes in naïve animals as shown by co-localization between GFAP immunostaining and EGFP transgene expression (Fig. 1e,f).

One of the unique features of AAV gene transduction is that it rarely integrates into the host genome. Following uncoating in the host nucleus, single-stranded genomes are converted to double-stranded multimeric circular concatemeric episomes^27^. As such, AAV transgene expression can persist long-term in post-mitotic cells. Because tumor cells undergo rapid cell division, it may be possible that transgene expression is lost over time through sequential dilution of episomes passed down to daughter cells. To explore this, we evaluated AAV6-EGFP transgene expression longitudinally across early time points in mice harboring RFP labeled GL261 cells, which demonstrate the highest transduction efficiency *in vitro* (**Extended Data** Fig. 1f). Tumors were resected 3, 5, and 7 days following AAV6-EGFP intra-tumor injection as outlined in Fig. 1g, and EGFP transgene expression in tumors and astrocytes was measured by flow cytometry. Even at early time points, AAV6 predominantly transduces astrocytes identified as GFAP + RFP− (70–80% EGFP + cells), with limited EGFP expression observed in RFP + tumor cells (< 15%) (**Extended Data** Fig. 1g, Fig. 4a). By 7 days post intratumor viral injection, less than 5% of EGFP + cells on average were RFP + tumor cells, where astrocytes consistently comprised 70–80% of EGFP + cells at each time point. These data indicate that AAV6 more selectively transduces astrocytes *in vivo*, with limited and transient expression in tumor cells.

### AAV6 transgene signal distribution and durability in GBM

**Figure 2.**
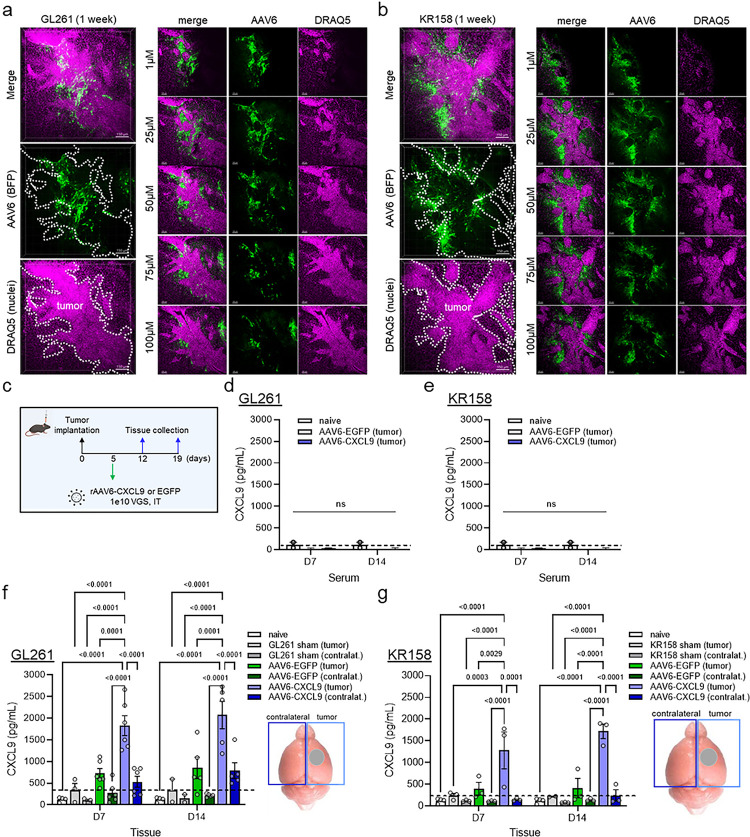
AAV6 tumor tropism. 3D IHC displaying geospatial distribution of AAV6 encoded transgene (BFP) in (a) GL621 and (b) KR158 tumors collected 1 week following *in vivo* transduction (green pseudocolor), n=2 per model. DRAQ5 nuclear dye (pink pseudocolor) is used to identify tumor borders, as outlined by the white dashed line. (c) Intra-tumor AAV6 treatment schematic for protein detection of AAV6 encoded CXCL9. ELISA detection of CXCL9 protein in serum extracted from peripheral blood draws at one and two weeks following AAV6-CXCL9 or AAV6-EGFP control intracranial injection in (d) GL261 and (e) KR158 models, n=3 per time point, per group. Age-matched naïve controls used to establish baseline CXCL9 levels indicated by dashed black line. ELISA detection of CXCL9 protein in brain tissues isolated at 1 and 2 weeks following AAV6-CXCL9 or AAV6-EGFP control intracranial injection in (f) GL261 and (g) KR158 models. Cerebellar tissue was removed and left and right hemispheres lysed separately to reflect tumor-bearing and contralateral (focal and distal) signal detection. Statistical analyses performed using two-way ANOVA analysis with Tukey’s multiple comparisons test. Age-matched naïve brain, and sham (saline) injected tumors included as negative control and tumor baseline control, with the latter represented by the dashed black line, n=3–6 per group with individual values shown. P-values = or < 0.05 are considered statistically significant.

Next, we examined the distribution of transgene signal in both the GL261 and KR158 tumor models via 3D IHC to better understand the avidity of AAV6 for tumor-associated versus distal astrocytes following direct intra-tumor injection. BFP transgene expression was observed in a peritumoral pattern in and around the tumor body in both model systems (Fig. 2a,b), redolent of glial scar formation found in human brain malignancies^28^. As CXCL9 is a small, secreted chemokine, we wanted to determine if signal expression was still focal to the tumor or could be detected in contralateral brain and/or systemically. The whole brain was collected at 1 and 2 weeks following AAV6-CXCL9 or AAV6-EGFP intratumor injection as outlined in Fig. 2c. Cerebellar tissue was removed, and remaining tissue was dissected into the tumor containing and contralateral hemispheres. Serum was collected following peripheral blood draws taken from the posterior vena cava. Brain tissue and serum were also collected from non-transduced (sham) tumor controls, and naïve (non-tumor bearing) controls to establish CXCL9 baseline values. Serum levels of CXCL9 following intratumor delivery of AAV6-CXCL9 measured using high sensitivity ELISA assay did not exceed those observed in naïve controls (Fig. 2d,e). In the brain, elevated CXCL9 expression was selectively detected in the tumor bearing hemisphere transduced with AAV-CXCL9, with minimal signal observed in the contralateral hemisphere in both GL261 and KR158 model systems (Fig. 2f,g). Transgene CXCL9 expression appears to be stable, as signal intensity was consistent in AAV6-CXCL9 transduced tumors at both the 1- and 2-week time points in each tumor model (Fig. 2f,g). Of note, a small increase in CXCL9 expression was observed in AAV6-EGFP control transduced GL261 tumors and could be indicative of a mild inflammatory response to AAV6, however these values were not found to be statistically significant. Together these data demonstrate that AAV6 intratumor delivery of CXCL9 results in focal and durable expression of encoded transgene, where tumor-reactive astrocytes are the target of AAV6 transduction.

### AAV6-CXCL9 enhances lymphocyte chemotaxis

**Figure 3.**
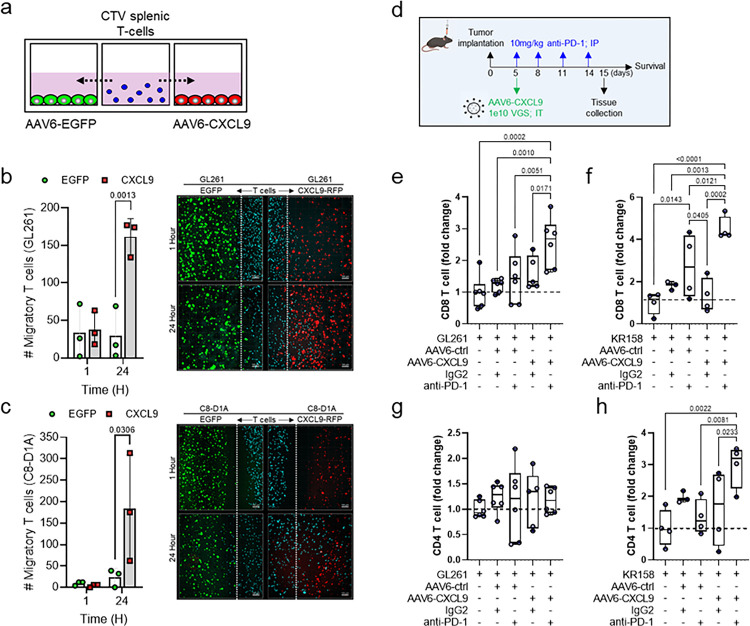
AAV6-CXCL9 directed lymphocyte chemotaxis. (a) Diagrammatic overview of *in vitro* competitive T lymphocyte chemotaxis assay. (b) Competitive chemotaxis measured as the number of T lymphocytes (CTV+, blue pseudocolor) in either AAV6-EGFP (control, green pseudocolor) transduced GL261 field or AAV6-CXCL9 (RFP+, red pseudocolor) transduced GL261 field at 1- and 24-hours following co-culture. Statistical analyses performed by two-way ANOVA with Sidak’s multiple comparisons test, n=3 per time point, per group. Representative images of competitive chemotaxis shown. Dashed white line represents the lymphocyte-tumor border at assay start. (c) Competitive chemotaxis measured as described in (b) in C8-D1A astrocytes field at 1- and 24-hours following co-culture. (d) Schematic outlining combination AAV6 and PD-1 ICB treatment and tissue collection and survival analysis in preclinical models. Multicolor flow cytometric detection of tumor-infiltrating CD8+ lymphocyte subsets in single or combination AAV6-CXCL9 plus anti-PD-1 ICB treatment in (e) GL261 and (f) KR158 models. AAV6-EGFP and IgG2 mAb control included as treatment controls. Fold-change normalization based on values detected in sham (saline) injected tumor control samples, with mean expression indicated by dashed black line. Statistical analysis performed using ordinary one-way ANOVA with Fisher’s least significant difference (LSD) test for multiple comparisons, n=3–6 per treatment group, individual values shown. Multicolor flow cytometric detection of tumor-infiltrating CD4+ lymphocyte subsets in single or combination AAV6-CXCL9 plus anti-PD-1 immune checkpoint blockade treatment in (g) GL261 and (h) KR158 models. AAV6-EGFP and IgG2 mAb treatment controls included. Fold-change normalization based on values detected in sham (saline) injected tumor control samples, with mean expression indicated by dashed black line. Statistical analysis performed using ordinary one-way ANOVA with Fisher’s LSD test for multiple comparisons, n=3–6 per treatment group, individual values shown. P-values = or < 0.05 are considered statistically significant.

To evaluate the biologic activity of AAV6-CXCL9 on lymphocyte recruitment, we performed competitive *in vitro* chemotaxis assays. Briefly, CTV-labeled splenic-derived T lymphocytes were flanked by target cells transduced with AAV6 encoding either EGFP or CXCL9, and migration was monitored via fluorescence microscopy at 1- and 24-hours following co-culture (Fig. 3a). Using GL261 tumor cells as the target population for AAV6 transduction, significantly more T lymphocytes co-localized in the CXCL9 transduced tumor field as compared to EGFP at 24 hours (Fig. 3b). Given that astrocytes are the principal target of AAV6 transduction *in vivo*, chemotaxis was reassessed via competitive co-culture using astrocytes (C8-D1A) in lieu of GL261 glioma cells. Astrocytes transduced with AAV6-CXCL9 similarly showed enhanced recruitment of T lymphocytes (Fig. 3c), confirming that transgene encoding CXCL9 produces a biologically functional chemokine. To determine the effect of AAV6-CXCL9 on T lymphocyte recruitment *in vivo*, multiparametric flow cytometry was performed to quantitate the number of T cells present in dissociated tumors following intratumor delivery. These studies were done in combination with anti-PD-1 ICB, where tissue was collected one day following the final dose of ICB to capture events within the therapeutic response window as outlined in Fig. 3d. In both GL261 and KR158 tumor models AAV6-CXCL9 alone had minimal impact on enhancing T cell recruitment to the tumor, however significant increases in T lymphocyte infiltration were observed in the context of combination treatment. AAV6-CXCL9 plus ICB increased CD8 T lymphocytes > 2.5-fold in the GL261 model and > 4.5-fold in the KR158 model (Fig. 3e,f and **Extended Data** Fig. 4b). While no significant changes in CD4 T lymphocyte recruitment in response to treatment was observed in the GL261 model (Fig. 3g), a > 3-fold increase was detected in the KR158 model (Fig. 3h). Anti-PD-1 ICB treatment in combination with control AAV6 (EGFP) modestly increased CD8 T lymphocyte recruitment in GL261 by 1.4-fold and in KR158 by 2.7-fold, indicating that CXCL9 markedly improves tumor infiltration by these cells. These data highlight a potential role for anti-PD-1 ICB in mobilizing T lymphocytes systemically, where sequestration of T lymphocytes was recently proposed as a novel mechanism of immune suppression in brain tumors^7^.

### AAV6-CXCL9 sensitizes preclinical GBM to anti-PD-1 ICB

**Figure 4.**
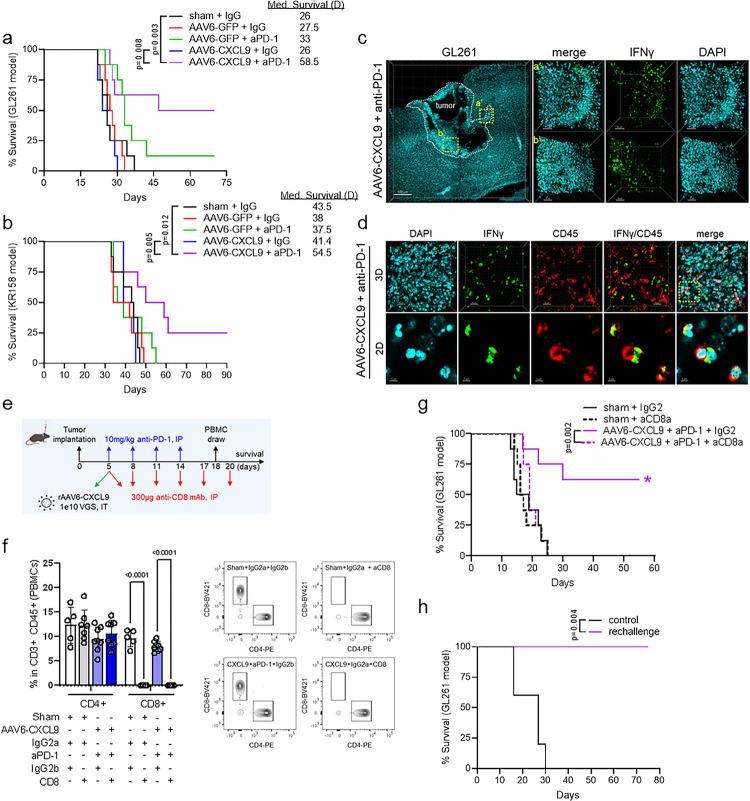
AAV6-CXCL9 sensitizes GBM tumors to anti-PD-1 immunotherapy. Survival analysis in (a) GL261 and (b) KR158 tumor-bearing mice treated with sham (saline) control, AAV6-CXCL9, and anti-PD-1 ICB alone and in combination. AAV6-EGFP and IgG2 are included as treatment controls. Median survival for each treatment group shown n=8 per group. Statistical analysis was performed using Log-rank (Mantel-Cox) test comparing individual treatment groups. (c) Tile-stitch 10× 3D IF imaging of GL261 tumors resected from combination AAV6-CXCL9 plus anti-PD-1 ICB treated GREAT mice. DAPI nuclear dye (blue pseudocolor) used to identify tumor area outlined by the dashed white line. (a’-b’) Digital magnification of regions outlined in the far-left panel to show higher image resolution. Green pseudocolor depicts endogenous EYFP, correlating with IFNγ expression. (d) 3D IF of tissue from GL261 tumor tissue as shown in (c) immunolabeled for CD45 expression (red pseudocolor). Digital zoom of region outlined in the far-right panel shows co-localization between CD45 and IFNγ, indicating these are immune cells. (e) Diagrammatic summary of combination treatment strategy with concomitant CD8α antibody depletion. (f) Flow cytometry detection of lymphocyte subsets isolated from peripheral blood (retro-orbital) collected at day 18 of study. Bar graph depicts the percent of each CD4 T lymphocytes (CD45+CD3+CD4+CD8−) and CD8 T lymphocytes (CD45+CD3+CD4−CD8+) detected within the total CD45+ population of PBMCs. Statistical analyses performed using Sidak’s multiple comparisons test, n=5–8 per group. Individual values shown. (g) Survival analysis in GL261 tumor-bearing mice treated with combination AAV6-CXCL9 plus anti-PD-1 monoclonal antibody, with or without anti-CD8α depletion. Sham (saline) injected GL261 tumors treated with anti-CD8α or control IgG2 included as treatment controls. Statistical analysis performed using Log-rank (Mantel-Cox) test comparing individual treatment groups, n=8 per group. (h) Survival analysis in long-term survivors from combination treated animals re-challenged with tumor at day 55 of study (n=5). Age-matched naïve control mice were orthotopically implanted with GL261 as survival control arm. Statistical analysis was performed using Log-rank (Mantel-Cox) test comparing individual treatment groups. P-values = or < 0.05 are considered statistically significant.

To assess if enhanced lymphocyte recruitment and immunological reprogramming through combination treatment could produce anti-tumor responses against GBM, we performed survival analyses in both the GL261 and KR158 syngeneic model systems. 5 days following tumor implantation, AAV6 encoding CXCL9 or EGFP control transgene was injected intratumorally, with anti-PD-1 ICB (10mg/kg) administered intraperitoneally for a total of 4 doses given every 72 hours (Fig. 3d). In the GL261 model we found that anti-PD-1 ICB produced a small, but non-significant increase in overall survival as compared to sham treated control animals (p = 0.060), where AAV6-CXCL9 treatment yielded no survival benefit as a monotherapy (Fig. 4a). Combination treatment significantly improved overall survival, with 50% of animals exhibiting durable outcomes (Fig. 4a). We observed similar results in the KR158 model, which carries a low mutational burden and is recalcitrant to immunotherapy, with combination treatment significantly improving median survival, and long-term survival observed in 25% of this cohort (Fig. 4b). As an additional metric to validate the ability of combination AAV6-CXCL9 plus anti-PD-1 ICB to immunologically transform GBM tumors, GL261 tumors were implanted in GREAT transgenic mice to evaluate tumor-wide IFNγ expression following treatment as described in Fig. 3d. IFNγ was detected in combination treated tumors evidenced by EYFP signal detection via 3D IHC (Fig. 4c). Immunolabeling of tissues for CD45 confirms that EYFP (IFNγ) + cells are immune cells (Fig. 4d), indicative of pro-inflammatory immune activation.

To determine if CD8 lymphocytes contribute to the therapeutic survival effect, we repeated combinatorial treatment with concomitant CD8 depletion (Fig. 4e) in the GL261 model. We found that on study day 18 all animals treated with CD8 depleting antibodies had no detectable levels of circulating CD8 T lymphocytes, and no changes in the quantity of circulating CD4 T lymphocytes (Fig. 4f, **Extended Data** Fig. 4b). CD8 depletion reversed the survival benefit observed with combination AAV6-CXCL9 plus anti-PD-1 ICB, and this cohort progressed as quickly as control treated subjects (Fig. 4g). To determine if combination treatment could confer long-term immune memory formation, we performed a GL261 tumor rechallenge in long-term survivors (> 55days) that had received AAV6-CXCL9 plus anti-PD-1 ICB. No observable residual tumor was present from the initial tumor implantation during the second implantation. A second cohort of age-matched naïve animals was intracranially injected with GL261 as a control. Control animals all succumbed to tumor burden within 30 days of tumor implantation, whereas 100% of rechallenge animals remained disease free (Fig. 4h). These data confirm that therapeutic response to combination therapy is dependent on tumor infiltration by CD8 T lymphocytes as part of the adaptive immune cascade, and combination therapy can convey long-term immune memory protection against recurrence.

### scRNAseq identifies treatment-related immune response to AAV6-CXCL9 and anti-PD-1 ICB

**Figure 5.**
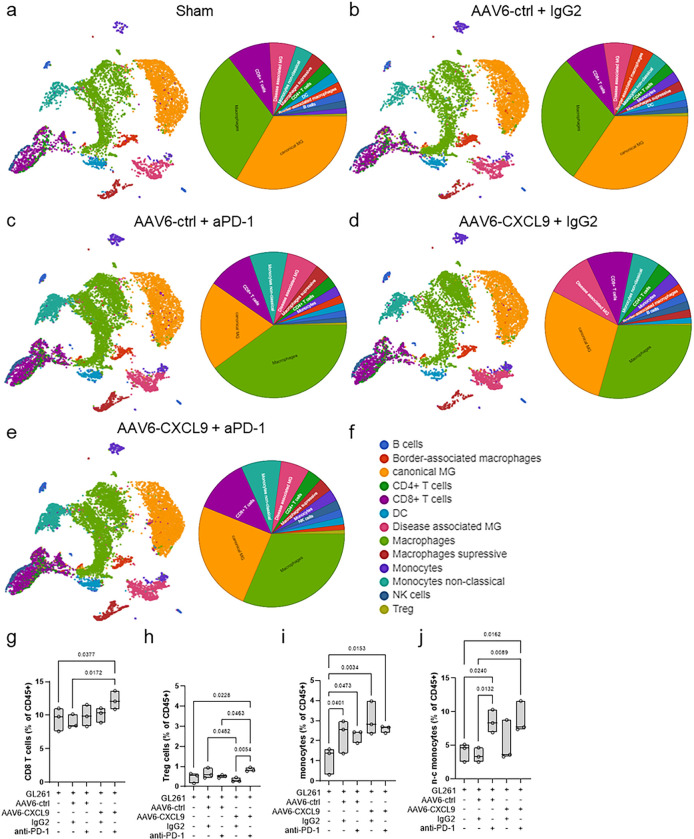
Immunological landscape of GBM tumors treated with AAV6-CXCL9 and anti-PD-1 immunotherapy. UMAP of cell types clustered by scRNA transcriptional analysis of 52,344 CD45+ cells isolated from GL261 tumor bearing mice treated with: (a) sham (saline), (b) AAV6-ctrl + IgG2, (c) AAV6-ctrl + aPD-1, (d) AAV6-CXCL9 + IgG2, and (e) combination AAV6-CXCL9 + aPD-1 treated GL261 tumors, n=3 per group. Summary circle chart depicting cell cluster population frequency detected for each treatment included alongside each UMAP. (f) Summary of UMAP cell clusters. Quantitative change in population frequency of (g) CD8+ T cells, (h) Treg cells, (i) monocytes, and (j) non-classical (n-c) monocytes across treatment groups. Statistical analyses performed using ordinary one-way ANOVA with Fisher’s LSD test for multiple comparisons, n=3 per group, individual values shown.

In an effort to define the immunological landscape of AAV6-CXCL9 treated tumors with or without concurrent anti-PD-1 ICB, we performed single cell RNA sequencing (scRNAseq) on CD45-positive cells isolated from GL261 tumors collected on day 15 of treatment as outlined in Fig. 3d. Dimensionality reduction using uniform manifold approximation and projection (UMAP) was performed on 52,344 cells collected across five treatment groups: sham (saline), AAV6-ctrl + IgG, AAV6-ctrl + aPD-1, AAV6-CXCL9 + IgG, and combination AAV6-CXCL9 + aPD-1, 3 mice per group (Fig. 5a–e).

Top differentially expressed genes from each pooled population were identified, and cluster cell types were defined using the expression of known marker genes resulting in the identification of 13 unique cell clusters^29,30^ (Fig. 5f **and Extended data Table 1**). Analyses of lymphocyte tumor recruitment across treatment groups recapitulate our earlier observation, with combination therapy yielding a significant increase in total infiltrating CD8 T lymphocytes (Fig. 5g), identified using the gene expression markers *Cd3d, CD8a, Cd8b1* as previously described^31^. T regulatory lymphocytes (Treg), defined by *Cd4, Foxp3,* and *Il2ra* gene expression^31^, were also increased in response to combination therapy, although collectively these represent < 1% of the total tumor-associated immune population (Fig. 5h). Increased tumor infiltration by monocytes, classified by high *Ly6c1* expression, was observed across all treatment groups as compared to sham control mice (Fig. 5i), with an enrichment of non-classical monocytes characterized by *Spn, Cx3cr1,* and *Tnfrsf1b* expression^32^ in groups receiving anti-PD-1 treatment (Fig. 5j). Graphical summaries for all remaining cells clusters in response to each treatment are shown in **Extended** Fig. 5a-h.

### Combination AAV6-CXCL9 and anti-PD-1 ICB treatment increases cellular crosstalk in lymphocytes

**Figure 6.**
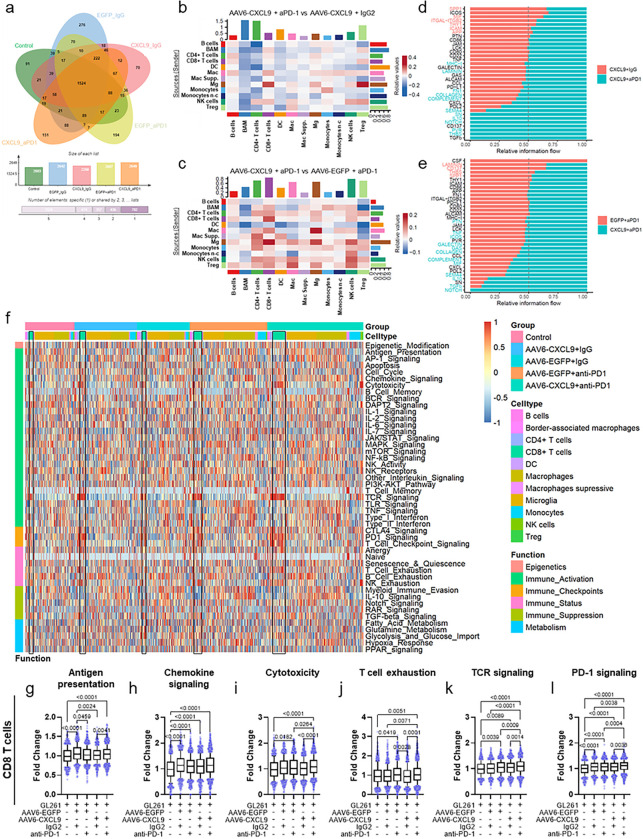
AAV6-CXCL9 and anti-PD-1 immunotherapy stimulates CD8 lymphocyte activation. (a) Venn Diagram representing differentially expressed genes affiliated with each treatment. (b) Heatmap depicting scRNA-seq-derived cell-cell communication networks enriched or decreased in response to combination AAV6-CXCL9 + aPD-1 as compared to AAV6-CXCL9 + IgG2 treatment across identified cell clusters. (c) Heatmap depicting scRNA-seq-derived cell-cell communication networks enriched or decreased in response to combination AAV6-CXCL9 + aPD-1 as compared to AAV6-EGFP + aPD-1 treatment across identified cell clusters. (d) Waterfall summary plot of scRNA-seq-derived signaling pathways enriched in CD8+ T cells following combination AAV6-CXCL9 + aPD-1 as compared to AAV6-CXCL9 + IgG2 treatment. (e) Waterfall summary plot of scRNA-seq-derived signaling pathways enriched in CD8+ T cells following combination AAV6-CXCL9 + aPD-1 as compared to AAV6-EGFP + aPD-1 treatment. (f) Heatmap representation of gene expression analysis derived from all cell clusters using the nCounter^®^ Immune Exhaustion Panel (nanoString) following AAV6-CXCL9 gene therapy with or without PD-1 ICB. CD8+ T cell populations outlined in black for each treatment group. (g-l) Quantification of common pathways found to be differentially regulated in CD8+ T cells in response to treatment. Statistical analyses performed using Kruskal-Wallis test followed by Dunn’s multiple comparisons, with individual values shown. P-values = or < 0.05 are considered statistically significant.

As shown in Fig. 6a, we identified 2,260 differentially expressed genes (DEGs) associated with AAV6-CXCL9 treatment, 2,607 DEGs associated with anti-PD-1 treatment, and 2,649 DEGs associated with these treatments combined. Of these, 70, 194, and 151 DEGs appear to be unique to each given treatment strategy, respectively, and may provide unique insight toward treatment impact on immune cell functional states. Through transcriptional expression of distinct ligands and receptors, cell-type-specific interactions were inferred, providing additional insight towards the inflammatory profile of tumors and how they change in response to treatment^33^. Using our predefined cell clusters, a simplified DEG set was established for each. DEGs were then queried against public ligand-receptor databases (see Methods). Summary results are shown in Chord Plots, where line thickness represents the number of predicted interactions between two defined cell clusters (**Extended Data** Fig. 6a-e). Next, we performed direct comparisons of interactome activity between treatment groups to elucidate heightened or decreased connectivity associated with AAV6-CXCL9 and anti-PD-1 ICB, where heatmap relative values in red indicate increases and blue decreases in prospective ligand-receptor interactions. In evaluating AAV6-CXCL9 in combination with either anti-PD-1 ICB or IgG2 control to resolve the contributions of ICB, notable increases in signals emanating from each macrophages (Mac), border-associated macrophages (BAM), microglia (Mg), and NK cells signaling to CD8 + and regulatory T cells were observed (Fig. 6b). Decreased incoming signals were noted in BAMs, CD4 + T cells, and dendritic cells (DCs) stemming from nearly all cell clusters (Fig. 6b). Cell-cell interactions associated with AAV6-CXCL9 shown in Fig. 6c reveal heightened communication directed toward both CD4 + and CD8 + T cell subsets, and NK cells prompted by all clusters excluding B cells and DCs. Signaling originating from all lymphocyte populations, and most innate immune cells including Macs, Mgs, Monocytes, and NK cells was increased, suggesting that AAV6-CXCL9 treatment broadly stimulates immune activity.

Given that combination treatment promotes CD8 T cell tumor infiltration, which is required for anti-tumor efficacy, we sought to resolve how treatment might impact CD8 T cell effector function via pathway analysis of DEGs specifically within these cells. Comparative pathway analysis between CD8 T cell DEGs shows selective enrichment of thrombospondin (THBS), poliovirus receptor (PVR, CD155), CD137 (4–1BB), fibronectin-1 (FN1), laminin, and major histocompatibility complex class I (MHC I), among others, as uniquely affiliated with combination therapy when compared to AAV6-CXCL9 plus IgG2 control (Fig. 6d). These data suggest that anti-PD-1 treatment prompts T cell activation via CD137^34^, but also reciprocal immune suppression via CD155 given its inhibitory function as a ligand for T cell immunoreceptor with immunoglobulin and ITIM domain (TIGIT)^35^. FN1, laminin, and THBS are major constituents of the extracellular matrix, and when produced by lymphocytes have been described to support cell-cell engagement, transendothelial migration, and lymphoproliferation^36–38^. DEG comparisons between combination therapy and AAV6-EGFP control plus anti-PD-1 ICB treatment reveals selective pathway enrichment of macrophage migration inhibitory factor (MIF), growth arrest specific (GAS), galectin, inducible T cell co-stimulator (ICOS), tumor necrosis factor (TNF), and pleiotrophin (PTN) as a result of AAV6-CXCL9 treatment (Fig. 6e). These data infer that AAV6-CXCL9 directly promotes CD8 T cell activation through increased ICOS and TNF expression^39,40^, T cell migration via PTN^41^, and reciprocally enhances innate immune stimulation of NK cells and myeloid cells via GAS and MIF secretion, respectively^42,43^. It also reveals galectin-9 as a possible mechanism for CD8 T cell acquired exhaustion^44,45^. Both anti-PD-1 ICB and AAV6-CXCL9 treatments stimulate NOTCH, TGFβ, IL-10, SEMA4, CXCL, Complement, and CCL pathway activation (Fig. 6d,e), each with varying impact on T cell maturation, effector function, homeostasis, survival, and migration^46–52^. Comparative pathway analysis was also performed for CD4 T cells across treatment groups (**Extended Data** Fig. 6f,g).

We next leveraged the NanoString nCounter^®^ Immune Exhaustion Panel to further characterize immune status and inflammatory signatures associated with each respective treatment. A summary of pathway activation across all cell subsets in response to individual treatments is shown in Fig. 6f and **Extended Data** Fig. 6h, with CD8 T cell clusters outlined in black for each treatment group. Through deeper analysis of differential pathway activation, specifically in the CD8 T cell subset, we found heightened antigen presentation (Fig. 6g), chemokine signaling (Fig. 6h), cytotoxicity (Fig. 6i), T cell exhaustion (Fig. 6j), TCR signaling (Fig. 6k), and PD-1 signaling (Fig. 6l). In particular, combination AAV6-CXCL9 plus anti-PD-1 ICB was associated with the highest increase in cytotoxicity, TCR signaling, and PD-1 signaling. Increased T cell exhaustion appears to be associated with AAV6-CXCL9 treatment. Given that monocyte tumor infiltration was additionally increased in response to treatment (Fig. 5i,j), we evaluated pathway activation in these cells to better understand their functional status. We found enhanced activation across 12 pathways, including antigen presentation, chemokine signaling, cytotoxicity, IL-10 signaling, JAK/STAT signaling, other interleukin signaling, T cell checkpoint, TGFβ signaling, TNF signaling, Type I interferon signaling, and Type II interferon signaling (**Extended Data** Fig. 7). Of these, AAV6-CXCL9 treatment appears to be associated with increased antigen presentation, cytotoxicity, JAK/STAT signaling, and Type I interferon signaling, where anti-PD-1 ICB induces IL-10 signaling, TLR signaling, and TNF signaling. Together these data are suggestive that treatment may augment the pro-inflammatory function of these cells.

### Cytokine profiling of combination AAV6-CXCL9 plus anti-PD-1 ICB

**Figure 7.**
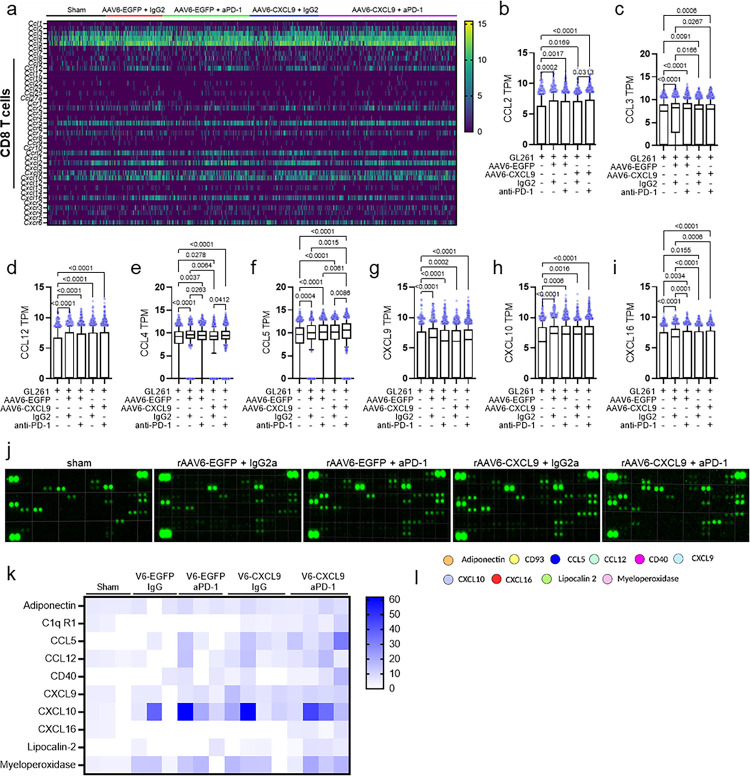
Inflammatory signature of preclinical GBM treated with AAV6-CXCL9 and anti-PD-1 ICB. (a) Heatmap summary of scRNA-seq-derived CCL-CXC expression in CD8+ T cells isolated from GL261 tumors in response to AAV6-CXCL9 and anti-PD-1 ICB treatment created using GraphPad Prism. (b-i) Quantification of CCL-CXC genes found to be differentially expressed in CD8+ T cells in response to treatment. Statistical analyses performed using Kruskal-Wallis test followed by Dunn’s multiple comparisons, with individual values shown. (j) Representative immunoblots depicting chemokine and cytokine protein expression detected in GL261 tumors resected following treatment with AAV6-CXCL9 with and without PD-1 ICB (n=3–4 per group). (k) Heatmap summary of CCL-CXC relative protein expression found to be differentially expressed in response to AAV6-CXCL9 with and without PD-1 ICB, created using GraphPad Prism. (l) Circos interactome analysis of detected differentially expressed proteins and predicted receptors. P-values = or < 0.05 are considered statistically significant.

As combination therapy increases DEGs of both the CCL and CXC superfamily of secreted chemokines and cytokines, we sought to parse out transcriptional changes within CD8 T cells as an additional means to evaluate the activation state of these cells given the central role of these ligands in directing migration and activation of immune cells during inflammation^53^. A summary of all CCL and CXC family ligand and receptor transcripts expressed by CD8 T cells is presented in the heatmap in Fig. 7a. CD8 T cell mediated stimulation of monocytes/macrophages is evidenced by increased transcription of CCL2, CCL3, and CCL12 across all treatment groups as compared to sham control (Fig. 7b–d). CCL4 transcription was also increased (Fig. 7e), indicative of NK stimulation by CD8 T cells. CCL5 was found to be the most differentially upregulated soluble ligand in combination treated CD8 T cells as compared to all other treatment groups (Fig. 7f), and is strongly indicative of CD8 T cell effector function^54,55^. While we show that AAV6 delivered CXCL9 transgene expression predominantly emanates from tumor-reactive astrocytes, our scRNAseq data shows that each anti-PD-1 and AAV gene therapy induces CXCL9 transcription within CD8 T cells (Fig. 7g), additionally demonstrating immune activation as a result of treatment^17,18,56^. CXCL10 was also found to be transcriptionally upregulated in response to anti-PD-1 and AAV gene therapy (Fig. 7h), which prompts further CD4, CD8, and NKT lymphocyte recruitment^56^. Altogether, these data support that combination AAV6-CXCL9 and anti-PD-1 ICB both increases lymphocyte trafficking to intracranial GBM tumors and potently stimulates effector lymphocyte cellular communication and activation.

As described above, secreted cytokines can influence the trajectory of tumors in a multitude of ways-reprogramming tumor-associated cells and suppressing infiltrating inflammatory subsets which allows for tumor tolerance, progression, metastasis, and even therapeutic resistance or, alternatively, creating an environment favorable for innate and adaptive immune activation to facilitate tumor rejection^57^. Moreover, the cytokine profile of a tumor may serve as predictive and/or therapeutic biomarkers allowing for the detection of tumor presence, forecasting therapeutic response, and can also be used to guide therapeutic choices^57^. We performed a large-scale cytokine proteomic assessment of single agent and combination treated tumors to identify candidate biomarkers of response to therapy. Tumors were resected ten days after the onset of treatment as shown in Fig. 3d. Of the 111 soluble murine proteins on the array, relative expression of 65 was detected in treated and/or control GL261 tumor samples as summarized **Extended Data** Fig. 8a, with representative cytokine immunoblots shown in Fig. 7j. 10 secreted factors were identified as differentially expressed as compared to sham control tumors following either single or combination treatment with AAV6-CXCL9 and anti-PD-1 ICB: ADIPOQ, C1QR1 (CD93), CCL5, CCL12, CD40, CXCL9, CXCL10, CXCL16, LCN2, and MPO (Fig. 7k, **Extended Data** Fig. 8b-k). Of these, CCL5, CD40, and CXCL16 were most potently induced by combination treatment. These markers are highly indicative of lymphocyte presence and activation, where CCL5 is a potent pro-inflammatory ligand manufactured principally by CD8 T lymphocytes, and CD40 is a co-stimulatory ligand that triggers lymphocyte proliferation and cytokine production ^54,55^. Of note, elevated CCL5 ligand expression demonstrates concordance with scRNAseq data (Fig. 7f). In addition, CXCL9, CXCL10 and CXCL16 are strong chemotactic signals for lymphocyte recruitment. Both CXCL10 and CXCL16 are induced by interferon gamma (IFNγ) and tumor necrosis alpha (TNFα), powerful catalysts of innate and adaptive inflammation^58,59^. A summary of treatment-induced secreted ligands and known receptor interactions are depicted via circular interactome analysis performed using Circos^®^ visualization software^60^, revealing insight towards immune reprogramming that occurs in response to each respective treatment (Fig. 7l). These data combined validate that AAV6 delivery of CXCL9 to the tumor microenvironment in tandem with anti-PD-1 ICB not only facilitate lymphocyte recruitment to GBM tumors, but also reprograms the immunological landscape towards a pro-inflammatory phenotype.

**Figure 8.**
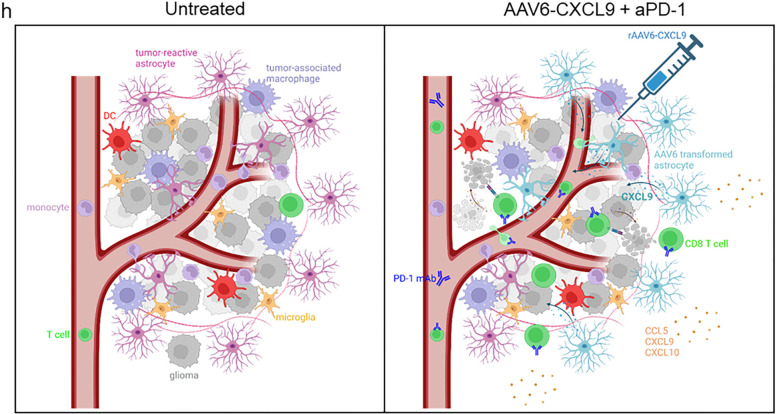
Diagrammatic summary of findings. Intra-tumor delivery of AAV6 encoding CXCL9 results in robust transduction of tumor-reactive astrocytes, creating a chemotactic gradient of secreted CXCL9. This improves lymphocyte trafficking in combination with anti-PD-1 ICB through chemokine-receptor engagement between CXCL9 in the TME and CXCR3 expression on lymphocytes. CD8+ T cells are required for durable survival response to treatment, indicating that tumor cell killing is mediated by the adaptive arm of immunity. Combination treatment also transforms the inflammatory milieu of tumors, creating a pro-inflammatory environment evidenced by the presence of cytokines and chemokines that further promote innate and adaptive immune activation. Created with BioRender.com.

In summation (Fig. 8), intra-tumor delivery of AAV6 encoded CXCL9 results in the production of a pro-lymphocyte chemotactic gradient by transduced tumor-reactive astrocytes. This, in concert with anti-PD-1 ICB, significantly increases tumor infiltration by lymphocytes likely through CXCL9 engagement with its cognate receptor expressed by these cells-CXC motif chemokine receptor 3 (CXCR3). In particular, CD8 T lymphocytes are the premier arbiters of anti-tumor response, where depletion of this lymphocyte subset negates therapeutic efficacy. Moreover, CD8 T cell effector activation and function is evidenced by heightened expression of co-stimulatory molecules, such as 4–1BB and ICOS, and production of pro-inflammatory chemokines and cytokines. Beyond CD8 T lymphocyte activation, AAV6-CXCL9 and anti-PD-1 ICB appear to contribute widespread immunological activation, demonstrated by heightened cellular cross-talk across numerous immune clusters, and protein detection of pro-inflammatory molecules. Notably, CCL5, CXCL9, CXCL10, and CD40 are detected in response to combination therapy within resected tumors, and may serve as biomarkers of therapeutic response.

## DISCUSSION

Perhaps one of the most consequential advantages of AAV gene therapy for the treatment of GBM, and possibly other solid tumors, is that fundamentally AAV is a modality for distributing encoded transgene into the tumor microenvironment. Vectors carrying unique biotherapeutic transgenes capable of targeting particular cells can be pooled, offering a straightforward method for delivering personalized anti-cancer combination treatment targeting one or multiple aspects of tumorigenicity. Examples include transgenes encoding anti-angiogenics, anti-migrastics, direct cytotoxic agents (e.g. suicide genes), immune stimulating elements, immune checkpoint inhibitor decoys, and even gene-editing elements such as CRISPR/Cas9 or shRNA. Herein, we demonstrate that AAV encoding for the lymphotactic chemokine CXCL9 can be leveraged to engage the immune system to recognize and attack tumor cells by modulating anti-PD-1 immunotherapy in the combinatorial setting.

We found that GBM tumors possess a chemokine signature that favors the recruitment of myeloid and other ‘suppressive’ immune cells, notably lacking lymphocyte call-and-receive signals. This is unsurprising given that the immune contexture of human GBM is largely comprised of myeloid cells^61^. These data suggest that deficiencies in lymphocyte trafficking and tumor infiltration likely contribute to this problem. To test if GBM reconstitution with a lymphocyte selective chemokine could improve trafficking, we leveraged AAV gene therapy to generate durable production of CXCL9. With the initial intent of transducing glioma cells to generate tumor-tropic transgene expression, we found that *in vivo* transduction was not redolent of *in vitro* screening methods, instead revealing potent transduction of tumor-reactive astrocytes with our lead serotype, AAV6. These findings advise caution in extrapolating AAV transduction efficacy from *in vitro* or even *ex vivo* screening methods to anticipated cell/tissue tropism in the *in vivo* setting. Despite this discrepancy, we found that AAV6-transduced astrocytes confer a high degree of tumor tropism, where transgene expression was limited to the immediate tumor area. This data suggests that tumor-associated astrocytes may be selectively susceptible to AAV6 transduction. Heparin sulfate proteoglycan (HSPG), one of the principal host cell receptors for AAV6 transduction^62^ is reported to be upregulated by astrocytes in response to brain injury ^25,26^, offering a possible explanation for the tumor-tropic nature of astrocyte transduction by AAV6.

AAV targeting of tumor-associated cells may carry several distinct advantages over directly targeting cancer cells. Given the genetic diversity of cancer cells, identifying a serotype that can reliably and consistently transduce tumor cells poses a challenge. Tumor-associated cells such as astrocytes, as demonstrated herein, or alternatively endothelial cells, microglia, etc. are more likely to have a homogenous genotype across patient tumors allowing for targeted off-the-shelf therapeutic development, drastically accelerating treatment timelines and reducing cost as compared to personalized medicine approaches necessary to guide serotype selection for cancer cell targeting. Our data also indicates that targeting of tumor-associated astrocytes is likely to produce a more durable response as these cells are less susceptible to genetic alterations. Direct tumor transduction, on the other hand, is short-lived, likely as a result of vector genome dilution due to the high proliferative capacity of these cells^63^ and may require repeat treatments to sustain transgene expression.

Although AAV6 produced CXCL9 clearly improves lymphocyte chemotaxis *in vitro*, we found that AAV6-CXCL9 monotherapy did not induce robust lymphotaxis into intracranial GBM tumors. The addition of anti-PD-1 ICB dramatically improved lymphocyte trafficking in the combinatorial setting with AAV6-CXCL9, in particular CD8 T cells. These findings speak toward the prospect of T cell sequestration as an auxiliary barrier to trafficking that jointly contributes to lymphopenia in GBM as first reported by Chongsathidkiet and colleagues^7^. Therefore, treatment strategies to boost peripheral lymphocyte counts may be necessary to realize the potential of AAV-based chemotactic therapy. Anti-PD-1 ICB offers one such modality, as peripheral expansion of T cells has been validated as a clinical correlate of response to this immune checkpoint inhibitor in certain cancers^64,65^. GBM is largely refractory to anti-PD-1 ICB including in somatically hypermutated GBM,^8,66^ reciprocally underscoring the need for multimodal treatment strategies. Our findings echo this sentiment, with anti-PD-1 ICB showing minimal improvements in overall survival in the preclinical setting. Another consideration is to combine AAV6-CXCL9 gene therapy alongside adoptive cellular transfer of *ex vivo* modified T cells, such as CAR T cell therapy. This strategy would bypass host lymphocyte sequestration altogether by direct intravenous delivery of antigen-specific T cells, where tumor-tropic CXCL9 expression would facilitate directed trafficking. Likewise, tumor-specific homing of CAR T remains an unresolved issue for solid tumors^9–11^ and may benefit from this particular combinatorial strategy. Both AAV6-CXCL9 and anti-PD-1 ICB also confer secondary mechanisms of action that protect against immune tolerance. CXCL9 is reported to promote lymphocyte differentiation and maturation towards an effector phenotype^67^ and anti-PD-1 ICB protects against TME PD-L1 induced lymphocyte exhaustion, thus contributing toward adaptive immune activation and prolongation of cytotoxicity in these cells^68^. Our findings corroborate this, confirming enhanced CD8 T cell cytotoxicity and TCR signaling pathway activation alongside detection of pro-inflammatory chemokines and cytokines associated with adaptive immunity, particularly in the context of combination treatment. Total abrogation of therapeutic efficacy with CD8 lymphocyte depletion validates adaptive immune activation as the principal mechanism of anti-tumor response. Combination virotherapy with pooled AAV vectors targeting different aspects of immunogenicity should also be explored further as a viable multimodal strategy for overcoming GBM immune evasion.

We found that direct intratumor delivery of AAV6 was sufficient to establish tumor-selective production of CXCL9. While intratumor delivery is considered an invasive method of treatment, generating focal expression of chemokine is critical for efficacy as the mechanism by which these secreted molecules work to facilitate immune cell trafficking is by establishing concentration gradients that immune cells expressing the cognate receptor follow, prompting selective infiltration into inflamed tissues^53^. While BBB-crossing serotypes have received significant attention for their novel ability to transduce CNS tissue following intravenous delivery, higher doses are required to maintain sufficient transduction efficiency, and off-target transduction of peripheral tissues remains a consequence of this delivery method^69–72^. Peripheral expression of CXCL9 or other lymphotactic chemokines could counterproductively deter homing to the CNS, reducing the efficacy of this treatment strategy. Furthermore, systemically delivered AAV could also encourage immunological responses resulting in host complement activation and antibody-mediated neutralization, or could prompt adverse toxicity such cytopenias, hepatoxicity, and even neurotoxicity^71–73^. To generate focal AAV transgene expression, virus could be delivered via stereotactic injection into unresectable gliomas, for example in tumors that arise in vital structures of the brain or in the event of recurrence, or into the resection cavity during surgery.

In summary, the use of AAV gene therapy has the potential to disrupt the existing treatment paradigm for GBM which relies on radiation, surgery, and cytotoxic chemotherapy. Systemic administration of immunotherapeutics and single-target chemotherapy agents have shown limited clinical efficacy due to dose-limiting toxicities, the constraints of the blood brain barrier (BBB), and the suppressive nature of the TME. This study combines the excellent safety profile of AAV^13^ with focal delivery directly to the TME, bypassing the restrictions and limitations of systemic delivery. AAV biotherapy is minimally invasive, tunable, and enables simultaneous delivery of multiple anti-cancer agents that can be customized to targets unique to each brain tumor. This platform has further application across multiple metastatic tumors where the TME limits the efficacy of immunotherapy.

## Methods

### Cell Culture

KR158B-luc (Kluc) glioma line (provided by Dr. Karlyne M. Reilly, NCI Rare Tumor Initiative, NIH) and GL261 cells have been verified histologically as high-grade glioma, and gene expression analysis confirmed appropriate haplotype background and expression of astrocytoma-associated genes^74^. CT-2A were purchased from Millipore Sigma. Primary human glioma cells including L0, L1, L2, CA1, CA2, CA4, CA6, CA7, L23, L26, L31, L34, L38, L47, and HA2 were a kind gift from Dr. Brent A. Reynolds^20^. C8-D1A primary astrocytes were purchased from ATCC. All cells were cultured in DMEM (Fisher-Scientific) supplemented with 10% FBS (VWR) and 1% Penn-Strep (Life Technologies), and maintained at 37°C in humidified conditions with 5% CO_2_. At the beginning of the study, cells were expanded, stocks made, and thawed vials were maintained in culture for no more than 3 weeks.

### *In vivo* studies

C57BL/6J (Strain# 000664), CCR2^RFP^CX3CR1^GFP^ (Strain# 032127), and GREAT (Strain# 017581) mice were purchased from Jackson Laboratory. On day 0, 5×10^4^ tumor cells suspended in 50% methylcellulose and 50% saline (Fisher-Scientific) were stereotaxically injected into murine brain at a depth of 3mm, 2mm lateral to bregma, at a volume of 2μl in 8–16-week-old animals. On day 5, AAV6 vectors were intratumorally injected in the same coordinates as tumor implantation. Where indicated, monoclonal antibody treatment (PD-1 ICB, IgG control, CD8a depletion) was administered beginning Day 5 via intraperitoneal injection and given every 72 hours. Protocols were reviewed and approved by the University of Florida Institutional Animal Care and Use Committee.

### Clinical Specimens

De-identified patient tissues were procured by the Florida Center for Brain Tumor Research (FCBTR) under the University of Florida Institutional Review Board protocols 201300482.

### Drug

InVivoMAb anti-mouse PD-1 and InVivoMAb rat IgG2a isotype control monoclonal antibodies were purchased from BioXcell, diluted in Sterile Saline 0.9% solution (Patterson Veterinary Supply, Inc.), and administered via intraperitoneal injection at a dose of 10mg/kg given every 72 hours for a total of 4 doses. InVivoMAb anti-mouse CD8α and InVivoMAb rat IgG2b isotype control monoclonal antibodies were purchased from BioXcell, diluted in Sterile Saline 0.9% solution (Patterson Veterinary Supply, Inc.), and administered via intraperitoneal injection at a dose of 300μg/mouse given every 72 hours for a total of 6 doses.

### AAV Protocol

HEK 293T cells (ATCC cat# CRL3216) were cultured to ~70% confluency in two Cellstacks (Corning cat# 3269) per construct and transfected using PEI 25k MW (Polysciences cat# 23966–1) for 3 days. The cells were then harvested via shaking and centrifugation until cell pellet was formed. The pellet was then digested with a final concentration of 50U/mL of Benzonase (Sigma cat# E8263) and 0.5% sodium deoxycholate in a lysis buffer (150mM NaCl, 50mM Tris-HCl pH 8.4) for 30 minutes at 37°C. Following incubation, the supernatant was supplemented with 5M NaCl until a 1M final concentration was achieved. Afterwards, the supernatant was lysed via 3 freeze thaw cycles of −80°C and 50°C. The lysate was spun down and supernatant transferred to an ultracentrifuge tube (Beckman cat# 342414), where it is layered with discontinuous layers of iodixanol (Accurate Chemical cat# AN1114542) to separate out viral particles from the supernatant. This was spun for 1 hour at 18°C at 69,000 rpm. The viral particles were isolated and removed, then washed four times in a dialysis column (Millipore cat# UFC910024) with PBS before being finally purified in a sterile filtration column (Millipore cat# UFC30DV00).

### AAV Quantification

The viruses were titrated by quantitative PCR (Bio-Rad CFX384) using custom probes designed to target the ITR sequences. First, 1 uL of the virus was treated with DNAseI (Thermo Fisher cat# 18068015) for 15 minutes at room temperature, inactivated by heat and EDTA, protein coat of virus digested with Proteinase K (Thermo Fisher cat# 25530049) and finished with a second heat-inactivation step. Following incubations, the sample was diluted and mixed with a Taqman PCR Master Mix (Thermo Fisher cat# 4352042) and the custom designed probes (Thermo Fisher cat# 4332078). The probe sequences were as follows: Forward –GGAACCCCTAGTGATGGAGTT, Reverse –CGGCCTCAGTGAGCGA, Probe – CACTCCCTCTCTGCGCGCTCG. The samples were then compared to a standard curve consisting of a linearized plasmid with ITRs from a range of 1e4 to 1e8 genomic copies per mL. The samples were then run on a standard program of 10 minute denature at 95°C, then cycled 39 times at 95°C at 1 minute and 60°C at 30 seconds.

### Lentiviral transduction of tumor cells

RFP labeled GL261 and KR158 tumor cells were transduced with a LentiBrite RFP Control Lentiviral Biosensor (Millipore-Sigma), MOI 50. Following cell expansion, RFP-positive cells were FACS sorted using a BD FACSAria-II cell sorter, yielding RFP-stable tumor cells.

### Proteome Arrays

Following resection, the right hemisphere (cerebellum removed) of murine brain (tumor-containing) were transferred to 1.5mL microtubes, snap frozen in LN2, and stored at −80°C until lysis. De-identified flash frozen patient GBM tissue was procured from the FCBTR. Tissue shavings were collected on dry ice and transferred to 1.5mL microtubes. 300–500μl PBS containing 1x Halt^™^ Protease/Phosphatase inhibitor (Thermo Fisher) and 1% Triton X-100 (Sigma) was added to samples and transferred to wet ice. Tissue was lysed manually using a 20-gauge needle attached to a 1mL syringe followed by vortexing every 5 minutes for 30–60 minutes. Supernatant was collected following centrifugation at 10,000G at 4°C, and assayed for protein concentration using a NanoDrop Spectrophotometer. 0.75mg of each human sample was used for the Human Chemokine Array Profiler (R&D Systems, ARY017), and 1mg of each murine sample was used for the Mouse XL Cytokine Array (R&D Systems, ARY028) following manufacturer’s instruction. Images were captured using BioRad ChemiDoc MP Imaging System with ImageLab 6.1 software over a series of exposure times. Mean voxel intensity per capture antibody was calculated using Imaris x64 v9.7.0, and protein signal was normalized against internal reference controls. Detected protein and predicted receptor interactions were analyzed and visualized using Circos^® 60^.

### ELISA

Tissue specimens were collected at 1 and 2 weeks post-AAV6 injection. Peripheral blood was taken from the anterior vena cava, centrifuged at 12,000rpm × 10 minutes @ RT, and serum collected and stored at −80C. Whole brain was resected, cerebellum removed, and divided into the tumor-bearing (AAV6 injected) and contralateral hemispheres. Naive brain and serum were collected and used to set the baseline for both week 1 and week 2 datasets. Tissue was snap frozen and stored at −80°C until lysis. Tissue was lysed using RIPA buffer containing 2x Halt protease/phosphatase inhibitor cocktail (Thermo Fisher) with manual dissociation performed using a 20-gauge needle attached to a 1mL syringe followed by vortexing every 5 minutes for 30–60 minutes, and maintained on ice. Following lysis, tissue samples were centrifuged at 12,000 rpm @ 4°C × 10 minutes. Supernatant was collected, and assayed for protein concentration using a NanoDrop. Protein concentrations were adjusted using RIPA buffer. MIG/CXCL9 ELISA (Thermo Fisher) performed according to manufacturer protocol. Serum diluted 1:2 using Assay Diluent B. Tissue sample concentration: 2mg. All samples run in duplicate.

### 3D Tissue clearing and immunolabeling

Brain tissue was collected after cardiac perfusion with cold saline followed by PBS supplemented with 4% acrylamide (Sigma-Aldrich), 0.05% N,N’-methylenebisacrylamide (Sigma-Aldrich), 4% paraformaldehyde and 0.25% VA-044 (TCI America). Tissues were stored at 4°C for 3 days to allow hydrogel permeation of tissues. Following hydrogel polymerization at 37°C ×3 hours, whole brain was sectioned to 2mm and passively cleared over 3–7 days with PBS containing 200mM boric acid (Sigma-Aldrich) and 4% sodium-dodecyl-sulfate (Fisher-Scientific), pH 8.5 at 50°C. After clearing, samples were washed in PBS with 0.1% Triton X-100 for 2 days, and immunostained at 4°C for 2 days with the following antibodies and stains: GFAP (Thermo Fisher, cat# PA1–10004), CD45 (Thermo Fisher, cat# 14–0451-82), anti-chicken Alexa Fluor^™^ 647 antibody (Thermo Fisher, cat# A-21449), anti-rat Alexa Fluor^™^ 568 (Thermo Fisher, cat# A-11077), and either DAPI (Sigma-Aldrich) or DRAQ5 (Thermo Fisher) nuclear dye. Samples were whole-mounted onto slides using 62% 2,2’-Thiodiethanol (Sigma-Aldrich). Images were acquired using a Nikon A1RMP confocal microscope and analyzed using Imaris x64 v9.7.0 software.

### Tissue Dissociation & Flow Cytometry

Brain tissue was digested using the Multi-tissue Dissociation Kit (Miltenyi Biotec) on a gentleMACS^™^ Octo Dissociator with heat, followed by sample clean-up using Debris Removal Solution (Miltenyi Biotec) according to manufacturer’s protocol. Tumor-infiltrating leukocytes were isolated using CD45 microbeads (Miltenyi Biotec) filtered through LS columns (Miltenyi Biotec) on a QuadroMACS Separator (Miltenyi Biotec) according to manufacturer’s protocol. Blood samples were collected from the anterior vena cava, and RBC lysis performed using Pharm Lyse solution (BD Biosciences) per manufacturer’s protocol. Samples were washed 2x with cold PBS. Unstained cells were reserved for unlabeled and FC controls, and dead cells were labeled with Zombie NIR^™^ Fixable Viability Kit (Biolegend) according to manufacturer’s protocol. Cells were washed 2x in PBS containing 0.5% BSA (Sigma) and 2mM EDTA (Thermo Fisher) FC buffer and blocked for 10 minutes on ice using TruStain FcX (Biolegend) prior to cell surface antigen labeling with the following antibodies: CD45-APC (Biolegend, cat# 103112), CD3-FITC (Biolegend, cat# 100204), CD4-PE (Biolegend, cat# 100408), CD8-BV421 (Biolegend, cat# 100738) for 45 minutes on ice. For astrocyte detection, cells were fixed and permeabilized using True-Nuclear^™^ Transcription Factor Buffer Set (Biolegend) following manufacturer’s protocol following debris removal step, with no CD45 microbead isolation. Samples were labeled with Zombie NIR^™^ Fixable Viability Kit (Biolegend), blocked with TruStain FcX (Biolegend), and immunolabeled with GFAP-APC (ThermoFisher, cat# 51-9792-82). Following immunolabeling, all samples were washed 2x with FC buffer and analyzed using a BD FACSymphony A3 flow cytometer.

### *In vitro* Chemotaxis

GL261 or C8-D1A cells were plated in 24-well dishes at 1×10^5^/well in pre-warmed complete media. AAV6-EGFP or AAV6-CXCL9 (RFP+) was added at a final concentration of 10^5^ VGS. 24 hours following transduction, cells were transferred into the outer chambers of μ-Dishes with 3-well culture inserts (Ibidi), 10^4^, suspended in 15μl of growth-factor reduced Matrigel^®^ (Corning). 40μl of complete media was added following polymerization for 10 mins at 37°C in humidified conditions with 5% CO_2_. CD3+ T cells were isolated from naïve C57BL/6 mouse spleen (8–12 weeks) using MojoSort CD3 T cell isolation kit (Biolegend) per manufacturer’s protocol. T cells were labeled with Cell Trace Violet dye (CTV) (Thermo Fisher) per manufacturer protocol. 1×10^4^ labeled T cells were suspended in 15 μl cold growth-factor reduced Matrigel^®^ (Corning), and added to the μ-Dish center well. Following polymerization as described above, media was removed from all wells, and 3-well insert was carefully removed. The gap between wells was filled with additional Matrigel to form a continuous substrate and allow for cell migration, and allowed to polymerize for 20 minutes. Complete media was added to cover cells, and incubated at 37°C in humidified conditions with 5% CO_2_. IF images were acquired using a Nikon A1RMP confocal microscope at 1- and 24-hours following co-culture, and T cell chemotaxis was quantified as the number of migratory cells visible in either the EGFP or CXCL9 (RFP+) transduced tumor/astrocyte field.

### Single Cell RNA Sequencing, Quality Control, and Data Analysis

Following whole brain resection, cerebellar tissue was removed and the right hemisphere collected for processing. Tissue dissociation and CD45 TIL isolation was performed as described under *Tissue Dissociation* above. The cells directly after depletion were washed with PBS and 0.04% bovine serum albumin two times and filtered with 40-μn cell drainer. Cells were collected by centrifugation at 500g for 5 min and subsequently counted with hemocytometer. Cells were diluted in ice-cold PBS containing 0.04% BSA at a density of 1000 cells/μL. The final cell suspension volume equivalent to 8000 target cells was used for further processing. Cells were loaded into a Chromium NextGEM Chip G (10x Genomics, Pleasanton, California) and processed in Chromium X following the manufacturer’s instructions. Preparation of gel beads in emulsion and libraries were performed with Chromium Next GEM Single Cell 3’ Kit v.3.1 (Dual Index) according to User Guide provided by the manufacturer. Libraries quality and quantity were verified with 2200 TapeStation (Agilent technologies, USA). Libraries were pooled based on their molar concentrations. Pooled library was sequencing on the NovaSeq 6000 instrument (Illumina, San Diego, California). For sequencing 3’ gene expression libraries we used following read length: Read 1–28 cycles; i7Index-10 cycles; i5Index-10 cycles; Read 2–90 cycles. Raw base call (BCL) files generated by NovaSeq 6000 sequencer were processed using Cell Ranger software (10X Genomics, version 7) for demultiplexing, barcode processing, and single-cell 3’-gene counting. Mouse genome reference GRCm38 was used for sequence alignment using STAR aligner. A read was considered exonic, if at least 50% of it mapped to an exon, intronic (if it was non-exonic and intersected an intron), or intergenic otherwise. For reads that aligned to a single exonic locus but also aligned to 1 or more non-exonic loci, the exonic locus was prioritized and the read was considered to be confidently mapped to the exonic locus. Cell Ranger also aligned exonic reads to annotated transcripts. An annotated transcript that aligned to the same strand was considered to be confidently mapped to the transcriptome. These confidently mapped reads were used for unique molecular identifier (UMI) counting and subsequent analysis to generate h5 files. The h5 file of each sample was then processed with Partek Flow analysis software (version 10). Cells meeting the following quality control (QC) parameters were included in the analysis: total reads between 1000 to 33,649; expressed genes between 187 to 5464; mitochondrial reads percentage <20%. Following this selection, we obtained 48159 cells that passed QC filters. Next, features were filtered in order to include only genes expressed in more than 0.01% of cells and 20,785 genes were retained. UMI counts were then normalized following Partek^®^ Flow^®^ recommendations: for each UMI in each sample the number of raw reads was divided by the number of total mapped reads in that sample and multiplied by 1,000,000, obtaining a count per million value (CPM), the normalized expression value was log-transformed. Starting from the normalized data node, we performed clustering analysis for each sample separately by means of graph-based clustering task in Partek^®^ Flow^®^ software which employs the Louvain algorithm. Clustering analysis was done based on the first 100 principal components. To visualize single cells in a two-dimensional space, we applied Uniform Manifold Approximation and Projection (UMAP) plot using the first 50 principal components for each sample separately and for the entire data set. Cell types were determined by the expression of marker genes that define specific cell types (Supplemental table 1). Pathway enrichment analysis for tumor cells and immune cells was performed with AUCell algorithm using the NanoString nCounter Immune Exhaustion panel. Interactions between immune populations were analyzed and visualized using the CellChat algorithm^33^. The pheatmap package was used for unsupervised hierarchical clustering to create heatmaps^75^.

### Statistical analysis

Statistical analyses performed using GraphPad Prism 9 as described in figure legends. Significance determined as p<0.05. Voxel-based co-localization was established using Imaris x64 v9.7.0 using the Coloc module with automatic threshold selection. For survival studies, animals were randomized prior to treatment.

## Data Availability

The data that support the findings of this study are available from the corresponding author upon reasonable request.
